# Flexible and Wearable Tactile Sensors for Intelligent Interfaces

**DOI:** 10.3390/ma18174010

**Published:** 2025-08-27

**Authors:** Xu Cui, Wei Zhang, Menghui Lv, Tianci Huang, Jianguo Xi, Zuqing Yuan

**Affiliations:** School of Integrated Circuits and Electronics, Beijing Institute of Technology, Beijing 100081, China; 1120223215@bit.edu.cn (X.C.); 1120220997@bit.edu.cn (W.Z.); 1120222239@bit.edu.cn (M.L.)

**Keywords:** tactile sensors, flexible sensing, multifunctional, social robots

## Abstract

Rapid developments in intelligent interfaces across service, healthcare, and industry have led to unprecedented demands for advanced tactile perception systems. Traditional tactile sensors often struggle with adaptability on curved surfaces and lack sufficient feedback for delicate interactions. Flexible and wearable tactile sensors are emerging as a revolutionary solution, driven by innovations in flexible electronics and micro-engineered materials. This paper reviews recent advancements in flexible tactile sensors, focusing on their mechanisms, multifunctional performance and applications in health monitoring, human–machine interactions, and robotics. The first section outlines the primary transduction mechanisms of piezoresistive (resistance changes), capacitive (capacitance changes), piezoelectric (piezoelectric effect), and triboelectric (contact electrification) sensors while examining material selection strategies for performance optimization. Next, we explore the structural design of multifunctional flexible tactile sensors and highlight potential applications in motion detection and wearable systems. Finally, a detailed discussion covers specific applications of these sensors in health monitoring, human–machine interactions, and robotics. This review examines their promising prospects across various fields, including medical care, virtual reality, precision agriculture, and ocean monitoring.

## 1. Introduction

Robotics has made significant progress in recent years, notably with the rapid development of social robots designed for interaction in various environments [1,2,3]. This evolution has created a high demand for advanced tactile perception systems, especially in sectors like service, healthcare, and industry. Effective and natural human–machine interaction is crucial across all their relative applications [4,5,6]. Among them, tactile perception serves as a core component that supports tasks involving object manipulation, navigation, and collaboration [7,8]. Tactile sensors allow robots to sense and respond to external stimuli, such as pressure, shear, and temperature, by mimicking human touch. Rigid tactile sensors often exhibit limited adaptability to curved surfaces and provide insufficient feedback in subtle detections. Rigid tactile sensors, based on carbon nanotube–epoxy nanocomposites for structural health monitoring [9], GNP/epoxy strain sensors [10], and CNT–epoxy adhesive joint monitors [11], demonstrate high mechanical stability but face inherent limitations in conforming to curved surfaces and detecting subtle stimuli. In contrast, flexible tactile sensors offer enhanced compliance, stretchability, and multimodal capabilities due to advancements in flexible electronics and materials. These are essential for recognizing actions such as handshakes or object handling. To date, researchers continue to seek breakthroughs in flexible tactile sensors, focusing on integrated design, improved sensitivity, and other key parameters [12,13,14,15,16,17,18]. However, current flexible tactile sensors still face challenges regarding integrated design and sensitivity improvements. They aim to conform to curved surfaces while delivering high-resolution feedback for optimizing performance in intelligent robots [19,20,21].

Integrating flexible tactile sensors into intelligent interfaces have great prospects. These sensors are typically embedded in a robot surface or wearable accessories, enabling the robot to naturally perceive tactile information from both the environment and users [22,23,24,25]. This is essential for robots acting in physical interaction with humans during indoor activities and fine manipulation tasks, such as grasping various objects and assisting with personal care. By providing real-time force feedback, these sensors can reduce damage risks to users or surroundings [26,27,28], and improve the overall interactive experience [29,30,31,32]. However, the use of flexible tactile sensors in robots faces challenges related in achieving high sensitivity, durability, and comfort. Minimizing power consumption is also important to support long operation and decrease burdens on power supplies and circuits. Moreover, ensuring compatibility with communication systems for seamless integration is also an emerging issue.

**Figure 1:**
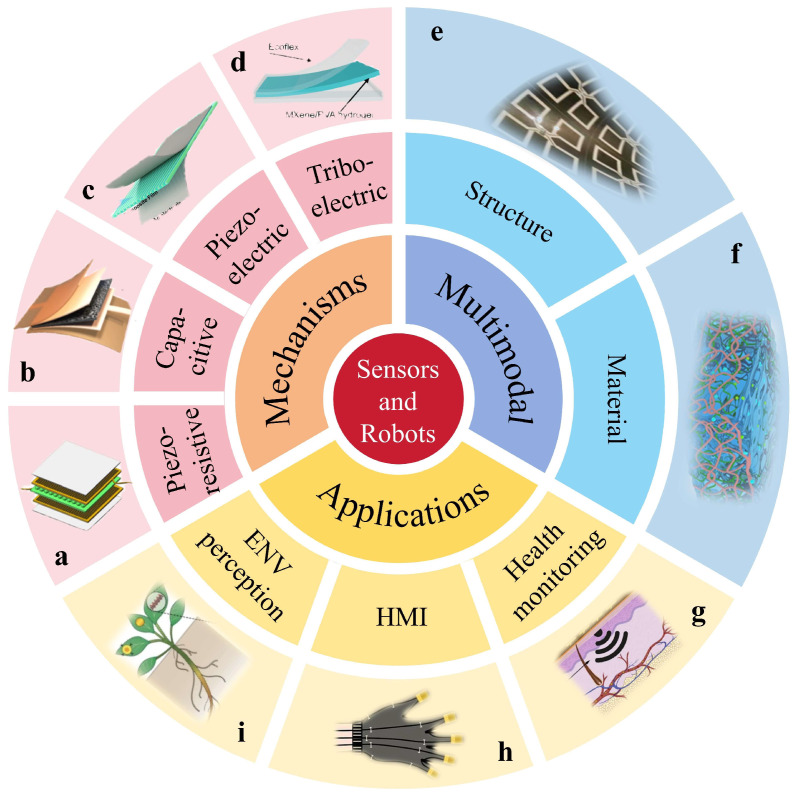
Schematic illustration of tactile sensors from mechanisms, multifunction, and applications. (**a**) Piezoresistive. Reprinted with permission from [33]. Copyright 2024 American Chemical Society. (**b**) Capacitive. Reprinted with permission from [34]. Copyright 2025 Elsevier. (**c**) Piezoelectric. Reprinted with permission from [35]. Copyright 2024 Springer Nature. (**d**) Triboelectric. Reprinted with permission from [36]. Copyright 2021 John Wiley and Sons. (**e**) Structure design. Reprinted with permission from [37]. Copyright 2024 Elsevier. (**f**) Material selection. Reprinted with permission from [38]. Copyright 2024 Elsevier. (**g**) Healthy monitoring. Reprinted with permission from [39]. Copyright 2022 Springer Nature. (**h**) HMI. Reprinted with permission from [40]. Copyright 2024 Elsevier. (**i**) ENV perception. Reprinted with permission from [41]. Copyright 2019 Springer Nature.

This review provides an overview of recent research on flexible and wearable tactile sensors, focusing particularly on their applications in intelligent interfaces. It discusses various transduction mechanisms used in tactile sensing, including piezoresistive, capacitive, piezoelectric, and triboelectric methods. The article also discusses advancements in multifunctional sensing technologies that simultaneously detect stimuli to enhance the replication of human tactile perception. Furthermore, this review details practical applications of flexible tactile sensors in health monitoring, human–machine interaction, and environmental sensing. The outline of this review is illustrated in the Figure 1. The main content emphasizes design and material consideration to improve sensor performance while addressing current technical challenges, which is aimed at systematically enhancing the capabilities of intelligent robots.

This review defines specific inclusion criteria for sources examined, ensuring coverage of key academic contributions. We adopted a systematic approach to literature retrieval, with primary searches conducted in the Web of Science Core Collection, Scopus, and IEEE Xplore databases, with supplementary searches in PubMed for biomedical applications, which offer a wide range of scientific journals. Relevant documents, such as review articles, research papers, and dissertations were systematically collected in January 2025. The search strategy combined key terms: (“flexible sensor” OR “wearable sensor”) AND (“tactile sensing” OR “haptic feedback”) AND (“intelligent interface” OR “human–machine interaction” OR “robotic perception”). Filters were applied based on publication year, paper type, publication format, and subject area. Initial searches yielded 2350 records published between 2018 and 2024. After duplicate removal, 1580 unique publications underwent two-stage screening. First, abstracts and titles were evaluated to exclude non-English publications, studies focused on rigid sensors, and purely theoretical investigations, retaining 620 records. Second, full-text assessment applied inclusion criteria emphasizing experimental validation with quantitative performance metrics (e.g., sensitivity, detection range, durability) and relevance to intelligent interfaces. This process excluded articles lacking empirical data or peer review, resulting in a final corpus of 128 high-impact publications, 83% of which have been published since 2020.

## 2. Basic Working Principles of Flexible Tactile Sensors

**Figure 2:**
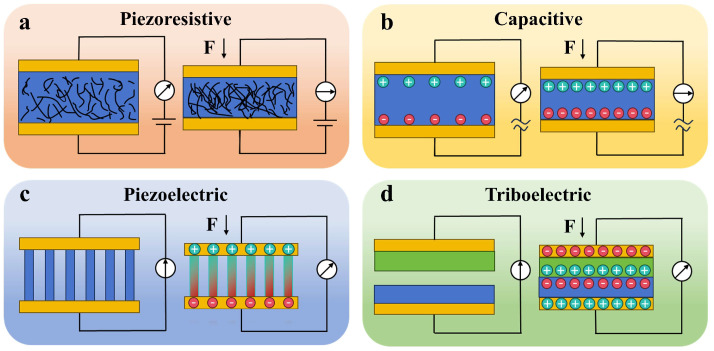
Schematic illustrations of the four basic working principles: (**a**) piezoresistive, (**b**) capacitive, (**c**) piezoelectric, and (**d**) triboelectric sensing.

Flexible tactile sensors perceive external mechanical stimuli, such as strain, pressure, shear, and twist, and convert these stimuli into electrical signals. Flexible tactile sensors can be classified into four types of piezoresistive, capacitive, piezoelectric, and triboelectric mechanisms. The mechanisms for each type of flexible tactile sensor are illustrated in Figure 2.

### 2.1. Piezoresistive Tactile Sensor

Piezoresistive tactile sensors operate on the principle that changes in electrical resistance are observable and measurable when a material deforms under mechanical stress [42,43]. These characteristics arise from the dynamic reconstruction of the microscale structure of the conductive materials. Specifically, the conductive pathways in the composite material consist of a network of filler particles (such as metal nanoparticles, carbon nanotubes, graphene flakes, or conductive polymers) dispersed within an insulating elastomer or polymer matrix. First, normal pressure or shear forces cause geometric deformations in internal conductive pathways, significantly altering overall resistance through contact points between nanoparticles, distribution of conductive fillers, or microstructure alterations within the percolating network. Second, the material resistance varies with external mechanical stimuli and is characterized by the relative change in resistivity to stress [44,45]. The formation of this conducting network depends on the percolation threshold, which is the critical concentration of packing required to create continuous conducting paths throughout the material. Achieving this threshold varies by filler type: high-aspect-ratio fillers, such as carbon nanotubes or nanowires, generally have lower percolation thresholds due to their ability to form networks with fewer contacts, while spherical particles or flakes tend to require higher concentrations for connectivity.

Piezoresistive tactile sensors have a simple structure, high sensitivity, easy integration, and low cost, making them suitable for flexible and wearable devices. However, creating cost-effective materials with high compressibility and sensitivity is still a challenge. The development of these sensors has led to various piezoresistive materials classified into three types: piezoresistive crystals, strain gauges, and composite materials [42]. Piezoresistive crystals, like ZnO and GaN, rely on crystal anisotropy, leading to varying resistivity under stress, as defined by piezoresistive coefficients [46,47,48]. Strain gauge-based sensors use metallic materials (e.g., Ag, Au), including nanoparticles and thin films. Their high conductivity allows them to function as an active sensor layer on deformable elastic substrates that change volume with strain, resulting in resistance variation. Data acquisition for piezoresistive sensors typically employs constant-voltage excitation with real-time current monitoring (e.g., using electrochemical workstations or LCR meters), or voltage divider circuits to measure resistance changes under mechanical stimuli, validated by subsequent experimental characterizations.

Composite materials consist of conductive fillers within a polymer matrix for flexibility and stability. There is a wide variety of conductive fillers, such as Ag, CNTs, MXene, graphene, and so on [49,50,51,52]. Common polymer substrates include polydimethylsiloxane (PDMS), Ecoflex, polyvinyl alcohol (PVA), polyurethane (PU), hydrogel, etc. [53,54,55,56]. The piezoresistive effect in composite materials arises from changes in the position and structure of conductive fillers caused by external forces, leading to alterations in resistance due to regenerating and reconfiguring conductive pathways. Composite materials are regarded as highly promising piezoresistive options because of their high sensitivity, cyclic stability, and diverse choices in material selection and structural design.

**Figure 3:**
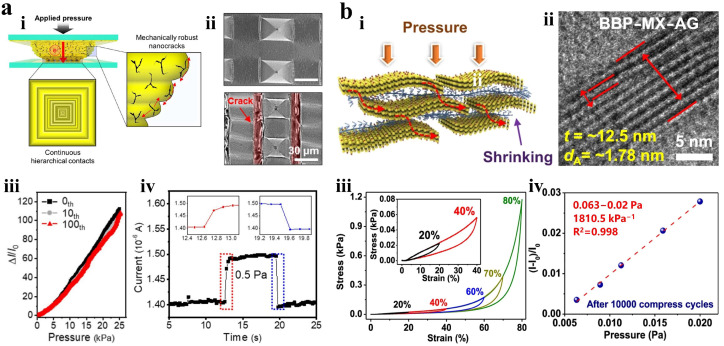
Piezoresistive tactile sensor. (**a**) (**i**) Schematic illustration of the piezoresistive pressure sensor. (**ii**) SEM images illustrate morphological changes on pyramid-structured PDMS. (**iii**) Pressure response curve after 100 tensile cycles at 30% strain. (**iv**) Response–recovery results under 0.5 Pa pressure. Reprinted with permission from [57]. Copyright 2024 American Chemical Society. (**b**) (**i**) Schematic illustration of conductive cross-linked MXene nanochannels. The arrow indicates the formation of a new conductive path. (**ii**) HRTEM images showing nanochannels with interlayer spacing of 1.8 nm. (**iii**) Compressive stress–strain curves of the sensor within 80%. (**iv**) Sensitivity curve of the sensor within 0.0063–0.02 Pa after 10000 compression–release cycles within 0–1 Pa. Reprinted with permission from [58]. Copyright 2022 Springer Nature.

Kim et al. proposed a soft piezoresistive pressure sensor with high-strain tolerance and linear sensitivity by using a thin nanocracked gold film (NC-GF) on polydimethylsiloxane (PDMS) as its electrode, as shown in Figure 3a(i) [57]. The uniformly distributed radial nanocracks in the Au electrode serve as sites for crack deflection and stress concentration, allowing cracks to open omnidirectionally. This configuration reduces the stress intensity and propagation rate at the crack tip, as shown in Figure 3a(ii). The sensor has a response time of 150 ms under a pressure of 0.5 Pa, as shown in Figure 3a(iii,iv). Shi et al. introduced a method to create soft polysiloxane crosslinked MXene aerogel featuring nanochannels in its cell walls for ultrasensitive pressure detection, as shown in Figure 3b [58]. The schematic illustration of the conductive path and a HRTEM image illustrate the exceptionally low detection limits of the sensor. The compressive stress–strain curve during compression–release cycles with up to 80% maximum strain showcases excellent mechanical performance. Additionally, this sensor exhibits extremely high linear sensitivity within a pressure range of 0.0063–0.02 Pa. Although the NC-GF/PDMS sensor achieves a linear response (Figure 3a), the use of precious metal electrodes considerably increases manufacturing cost, and the response time of 150 ms remains insufficient for dynamic contact scenarios, such as robotic grasping. In contrast, the ultra-low detection limit (0.0063 Pa) of MXene aerogel highlights the benefits of its nanochannel structure, but this two-dimensional material suffers from poor environmental stability.

**Figure 4:**
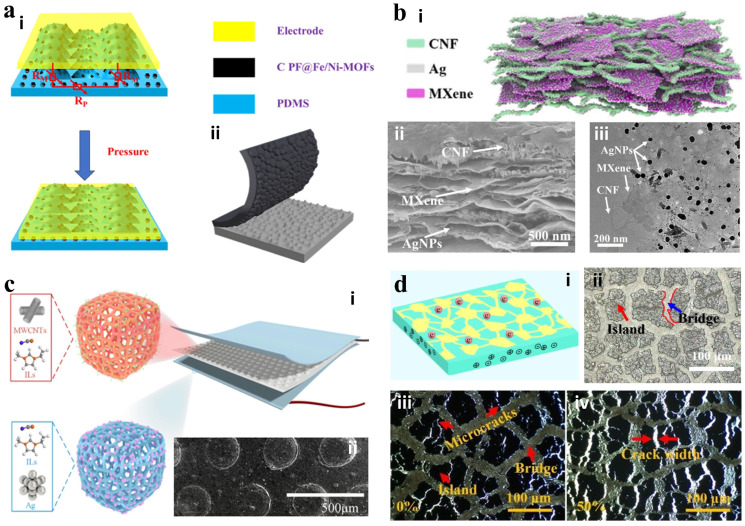
Microstructure of piezoresistive sensors. (**a**) (**i**,**ii**) Schematic illustration of the C PF@Fe/Ni-MOFs-based sensor. Reprinted with permission from [63]. Copyright 2025 Elsevier. (**b**) (**i**) Schematic illustration of the AMC composite. (**ii**,**iii**) Cross-sectional SEM images and TEM images of the AMC composite. Reprinted with permission from [64]. Copyright 2024 Elsevier. (**c**) (**i**) Schematic illustration of the porous ILs-MWCNTs-PU sensor. (**ii**) SEM images of the sensing layer. Reprinted with permission from [65]. Copyright 2024 American Chemical Society. (**d**) (**i**) Schematic diagram of the PAM/SA with conductive FCNTs coating. (**ii**) Photo of the island-bridge FCNTs conductive coating. (**iii**,**iv**) In situ optical imaging displays microstructural changes under 0 to 50% tensile strain. Reprinted with permission from [66]. Copyright 2024 Elsevier.

To improve sensor performance, detection range, and sensitivity, various nano-microstructures have been proposed for sensor preparation, such as microscale porous structures [59], pyramids [60], hemispheres [33], and columns [61,62]. Han et al. carbonized 2-aminoterephthalic acid iron–nickel metal–organic framework (Fe/Ni-MOF) and added phenolic resin (PF) to enhance electrical conductivity [63]. The C PF@Fe/Ni-MOF was dissolved in xylene and uniformly sprayed onto sandpaper. PDMS was then quickly coated over the sandpaper. The C PF@Fe/Ni-MOF diffused to the PDMS surface, creating a unique structure aligned with the sandpaper texture, as shown in Figure 4a. This distinct structure exhibits a sensitivity of 198.51 kPa^−1^. Although the sandpaper-like texture microstructure of carbonized MOF significantly improves sensitivity, its rigid carbon skeleton is prone to microcracks under repeated compression, resulting in an irreversible increase in contact resistance that limits the long-term reliability of dynamic pressure monitoring. Yang et al. fabricated a conductive hybrid Ag@MXene-CNF (AMC) film made of MXene, cellulose nanofibers (CNFs), and silver nanoparticles (AgNPs) using a vacuum filtration method to create a three-dimensional interconnection network, as shown in Figure 4b [64]. The AgNPs on the AMC surface enhance electrical conductivity and increase surface roughness, thereby expanding the contact area. Consequently, the AMC-based pressure sensor shows a sensing range of 0.098 to 9.8 kPa and achieves excellent pressure sensing capabilities due to its conductivity and microstructural features.

Sun et al. designed a highly flexible and high-performance piezoresistive sensor, as shown in Figure 4c [65]. The porous PU was doped with silver nanoparticles and incorporated ionic liquid (IL) as a flexible electrode layer. A convex microarray patterned sensing layer was fabricated by using heterogeneous ILs-MWCNTs-PUs formed by stainless steel mesh molding. The sensor exhibits a sensitivity of 7.023 kPa^−1^, pressure detection ranges up to 420 kPa, a sensing limit of 0.1 Pa, operational stability exceeding 80,000 cycles, and a fast response/relaxation time of 60 ms/80 ms. Zhang et al. developed a polyacrylamide/sodium alginate (PAM/SA) hydrogel as a flexible substrate [66]. Island-bridged microstructured functionalized carbon nanotubes (FCNTs) were created via secondary chemical cross-linking and ionic cross-linking. This approach resulted in an electron-conducting network with strong interfacial bonds, as shown in Figure 4d. These conductive hydrogels combine the flexibility of traditional hydrogels with the high conductivity of FCNTs. With a dual conductive percolation network, these organic hydrogel-based strain sensors show high sensitivity (strain factor GF = 76.54) and a wide operating range (0–600% strain). Although the covalent/ionic double cross-link design of island-bridge microstructured hydrogels enhances stretchability, its hydrophilic group is prone to non-uniform swelling in a hypertonic environment, resulting in unstable impedance response.

### 2.2. Capacitive Tactile Sensors

The capacitance (C) is influenced by the distance between electrode plates, overlap area, and dielectric material. Capacitive sensors detect mechanical stimuli via changes in capacitance [67]. Basic capacitive sensors use a metal film as an electrode with an elastomer dielectric layer between two electrodes. Pressure affects the distance, while shear force impacts the overlap area. In practice, a shear force applied parallel to the electrodes causes lateral displacement or deformation. This motion disrupts the relative alignment of the electrode plates, thereby reducing their effective overlap area and capacitance. These sensors are characterized by high sensitivity, resolution, fast response time, and low power consumption. The performance of flexible capacitive sensors can be improved by altering the dielectric material, modifying their structure, or integrating materials with a high dielectric constant [68,69,70]. Researchers are currently advancing high-performance capacitive sensors using designs such as microporous layers, microcolumns, and micro-pyramids to enhance sensitivity and detection range. Data acquisition for capacitive sensors can employ LCR meters to directly measure capacitance changes under applied stimuli, as experimentally validated in subsequent sensor implementations.

**Figure 5:**
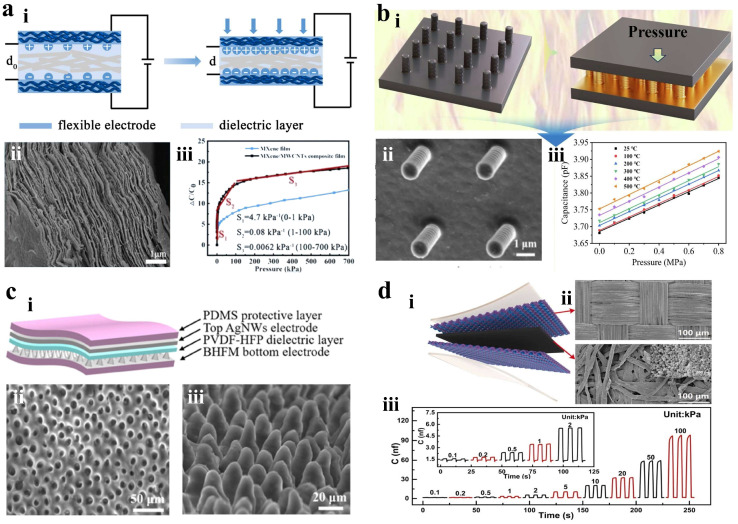
Capacitive tactile sensors. (**a**) (**i**) Cross-sectional SEM image of MXene/MWCNT composite film. (**ii**) Sensitivity comparison of the pressure sensor. (**iii**) Schematic working mechanism of the capacitive pressure sensor. Reprinted with permission from [71]. Copyright 2024 Elsevier. (**b**) (**i**) Capacitive pressure sensors based on microstructured SiCN ceramics. (**ii**) SEM image of the column arrays. (**iii**) Output capacitance as a function of pressure at different temperatures. Reprinted with permission from [72]. Copyright 2025 Elsevier. (**c**) (**i**) Schematic structure of the sensor. (**ii**) SEM images from a top-down perspective of microstructure caves. (**iii**) SEM images from a 45° tilt perspective of the BHFM. Reprinted with permission from [73]. Copyright 2024 American Chemical Society. (**d**) (**i**) Schematic structure of the sensor. (**ii**) SEM images of the polyester fiber conductive tape and the ink filter paper. (**iii**) Response and recovery curves of the sensor from 0.1 to 2 kPa. Reprinted with permission from [74]. Copyright 2024 Elsevier.

Cui et al. fabricated a capacitive pressure sensor by incorporating MWCNTs into MXene to improve electrode conductivity, as shown in Figure 5a [71]. The electrodes are obtained through filtering MXene and MXene/MWCNTs solutions and feature a distinct layered structure that enhances detection range and sensitivity. Additionally, biodegradable cellulose paper serves as the dielectric layer. The resulting sensors demonstrate high sensitivity of 4.7 kPa^−1^ for low pressures from 0 to 1 kPa and a broad detection range of 0–700 kPa. Ma et al. designed a SiCN-based capacitive pressure sensor with a microcolumn structure and high temperature resistance, as shown in Figure 5b [72]. The sensors exhibit low hysteresis, nonlinearity of 0.26%, and excellent repeatability at room temperature. At 500 °C, the sensors maintain stable performance with a sensitivity of 0.214 pF MPa^−1^, with oxidation/corrosion resistance and thermal stability. Although SiCN ceramics exhibit excellent temperature resistance up to 500 °C, overcoming the intrinsic thermal limitations of polymers, their sensitivity (0.214 pF·MPa^−1^) is less than 1/100 of that of room-temperature polymer-based devices. In addition, the brittleness of micro-column arrays results in poor installation fault tolerance, restricting their suitability for industrial applications.

Lan et al. developed a high-performance flexible capacitive pressure sensor featuring biomimetic hibiscus flower microstructures (BHFM) as electrodes and an ionic gel film dielectric layer [73]. The BHFM electrodes, fabricated via a simple, low-cost templating method, offer advantages over traditional microstructures (e.g., pyramids). The ionic gel boosts interface capacitance via electric double layer (EDL) formation. The sensor achieves ultrahigh segmental sensitivity (0–1 kPa: 48.57 kPa^−1^, 1–30 kPa: 15.24 kPa^−1^, 30–120 kPa: 3.74 kPa^−1^), fast response (<58 ms), low detection limit (5.5 mg), and excellent stability (>3000 cycles). It successfully monitors subtle physiological signals (e.g., tri-peak pulse waveforms, vocal vibrations, breathing) and higher pressures (e.g., plantar pressure), demonstrating significant potential for healthcare monitoring and human–machine interfaces. Li et al. achieved high-performance capacitive pressure sensors by integrating surface microstructures and enhancing the dielectric constant, as shown in Figure 5d [74]. Polyester fiber conductive tape serves as the electrodes while rough filter paper acts as the dielectric layer. This roughness increases the electrode–dielectric gap for improved performance. Daily carbon ink is infused into filter paper to boost its moderate conductivity. This sensor offers a wide detection range from 0.1 to 100 kPa, with high sensitivity of 1.47 kPa^−1^ between pressures of 0.1 and 10 kPa, along with excellent durability. The MXene/MWCNTs electrode has a wide detection range (0–700 kPa) but low sensitivity (4.7 kPa^−1^) [71]. In contrast, the BHFM ion gel sensor achieves an ultra-high sensitivity of 48.57 kPa^−1^, but its microstructure irreversibly collapses exceeding 100 kPa [73]. This highlights the inherent trade-off between achieving high sensitivity and maintaining a broad detection range.

### 2.3. Piezoelectric Tactile Sensor

Piezoelectric tactile sensors utilize the piezoelectric effect found in non-centrosymmetric crystals. When mechanically stimulated, these materials generate charge and voltage through internal polarization, converting mechanical energy into electrical energy [75]. By measuring the generated charges and voltages, one can determine the magnitude and distribution of external forces [76]. Common piezoelectric materials include polymers like polyvinylidene fluoride (PVDF), inorganic materials (BaTiO_3_, PZT, ZnO, PbTiO_3_), and composite materials. These sensors demonstrate high sensitivity and self-powering capabilities. For instance, PZT-polymer and PVDF-SiC composites improve dielectric properties and mechanical strength [77,78,79,80,81], which are significant for complex structures. Data acquisition for piezoelectric sensors requires high-impedance instruments (e.g., Keithley 6514 electrostatic meter) to accurately capture transient voltage and as-produced charges under mechanical stimuli, with output characteristics validated in subsequent experimental implementations.

**Figure 6:**
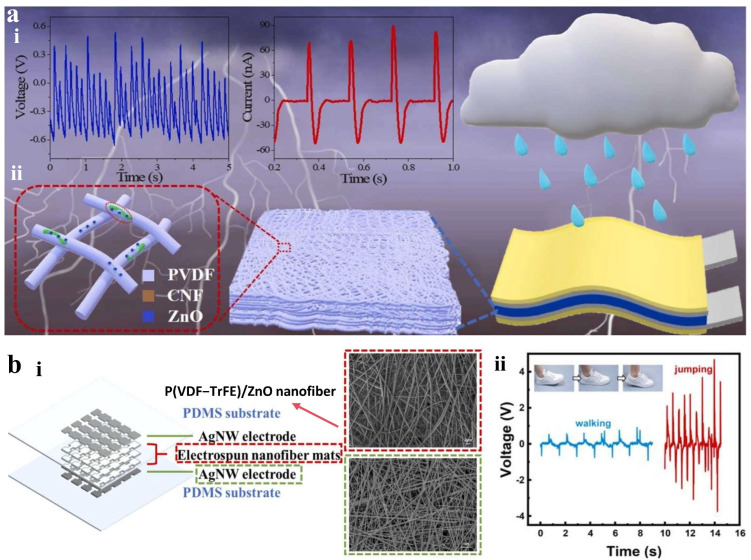
Piezoelectric tactile sensor. (**a**) (**i**) Voltage signals from water droplets falling on the PVDF/CNF@ZnO sensor. (**ii**) Schematic illustration of the sensor structure and CNF@ZnO PVDF nanofibers. Reprinted with permission from [82]. Copyright 2024 Elsevier. (**b**) (**i**) Schematic illustration of the multilayer P(VDF-TrFE)/ZnO piezoelectric sensor and SEM images of the nanofiber and AgNW electrode. (**ii**) The output voltage of the sensor. Reprinted with permission from [83]. Copyright 2024 Elsevier.

Zhu et al. developed a large-area flexible PVDF/CNF@ZnO composite film via electrospinning, utilizing a modified two-step hydrothermal method to synthesize ZnO-loaded cellulose nanofiber (CNF@ZnO) composites dispersed in polyvinylidene fluoride (PVDF) [82]. As illustrated in Figure 6a, the CNF@ZnO composite effectively increased the β-phase content of PVDF to 87.36%, enhanced the piezoelectric response to 24.65 pm·V^−1^, and achieved a longitudinal piezoelectric coefficient (d_33_) of 31 ± 2.07 pC·N^−1^. The composite film demonstrated remarkable mechanical properties with a tensile strength of 16.12 ± 2.35 MPa and an elongation at break of 16.21 ± 2.17%. The sensor generated approximately 1.68 V output voltage under water droplets. The tensile strength of the PVDF/CNF@ZnO is only 35% of that of P(VDF-TrFE)/ZnO composite (45 MPa) [83], and the improvement of piezoelectric properties comes at the expense of mechanical flexibility. Chen et al. fabricated a multilayer piezoelectric sensor array using enhanced electrospinning of poly(vinylidene fluoride trifluoroethylene) P(VDF-TrFE)/ZnO, as shown in Figure 6b [83]. P(VDF-TrFE) provides superior piezoelectric properties and high energy conversion efficiency. The electrospinning process operates under a high-voltage electric field to achieve fiber stretching and in situ polarization, significantly simplifying the process. The resulting nanofiber mat features high β phase content and an extensive specific surface area. Incorporating ZnO nanoparticles into P(VDF-TrFE) improves the piezoelectric effect while serving as nucleating agents that promote β phase formation, with an optimal concentration of 15 wt% maximizing β-phase content (82.04%) and piezoelectric output (9.44 V). Beyond this, ZnO agglomeration at 20 wt% reduces the stretchability of fiber, β-phase crystallinity, and mechanical strength, leading to declined performance. This sensor employs composite nanofibers as sensitive layers for pressure monitoring and location sensing. But the contact resistance at the electrode–nanofiber interface increases with repetitive bending, indicating interfacial reliability issues that limit the long-term stability of such flexible piezoelectric devices.

### 2.4. Triboelectric Tactile Sensor

Triboelectric tactile sensors operate based on the contact electrification and electrostatic induction. They involve two materials with different electron affinities coming into contact and sliding against each other, leading to static charge transfer that generates opposite charges at their interface [84]. Upon release, charged surfaces separate while electrostatic induction creates compensatory charges in the electrode, resulting in a voltage difference between electrodes [68]. An applied external force triggers the triboelectric effect, producing an electrical signal for detecting pressure, touch, and vibration. These sensors can utilize various materials, including polymers, metals, fabrics, and composites by incorporating conductive or dielectric nanomaterials, which enhance sensitivity and optimize structural design to improve sensing performance [85,86,87]. Data acquisition for triboelectric sensors requires high-precision electrostatic meters (e.g., Keithley 6514) to capture transient voltage/current signals generated during contact-separation cycles, with the measured output characteristics experimentally validated in subsequent implementations.

**Figure 7:**
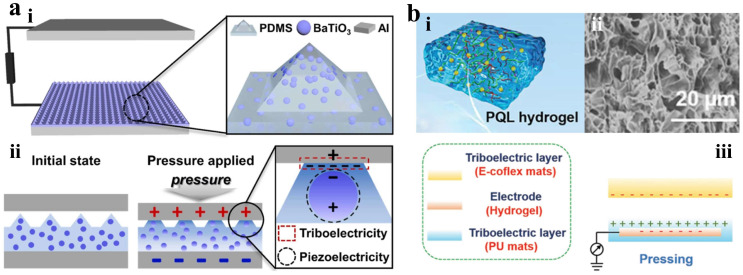
Triboelectric tactile sensor. (**a**) (**i**) Schematic illustration of BaTiO_3_ nanoparticle-embedded TENG. (**ii**) Working principle of TENG. Reprinted with permission from [88]. Copyright 2024 Elsevier. (**b**) (**i**) Schematic illustration of the hydrogel. (**ii**) SEM images of the hydrogel. (**iii**) Schematic working principle of the TENG-based sensor. Reprinted with permission from [89]. Copyright 2022 John Wiley and Sons.

Seong et al. designed a high-performance sensor that utilizes triboelectric–piezoelectric principles by incorporating BaTiO_3_ nanoparticles into pyramid-shaped PDMS elastomers, as shown in Figure 7a [88]. The mechanical deformation of PDMS and the polarization of BaTiO_3_ work together to generate triboelectric–piezoelectric charges on the surface. The pyramid-shaped microstructure significantly enhances mechanical deformation and sensitivity, increasing contact area and improving sensor performance. The pressure sensitivity reaches 3.71 V kPa^−1^ over a sensing range of 0.1–100 kPa, paving the way for effective conversion of mechanical actions into electrical signals. Yu et al. developed a stretchable conductive hydrogel for a high-performance triboelectric nanogenerator (TENG) sensor, as shown in Figure 7b [89]. Highly cross-linked hydrogels were created by chemically linking polyacrylamide and lithium magnesium silicate, enhanced with carbon quantum dots. Lithium magnesium silicate provides robust covalent bonds, improving the mechanical properties of the hydrogels. This hydrogel-based strain sensor demonstrates excellent sensitivity across a wide strain range and functions as a flexible triboelectric electrode for detecting pressures between 1 and 25 N, generating a short circuit current of 2.6 µA and an open circuit voltage of 115 V. The 115 V high-pressure output of the carbon quantum dot-reinforced hydrogel relies on its ionic conductive network. However, ion mobility decreases with temperature, leading to a 35% reduction in output from 25 to 45 °C, highlighting the urgent need to improve the thermal stability of ionic triboelectric materials.

Table 1 systematically compares the core performance characteristics and material properties of the four types of tactile sensors. While each category exhibits unique advantages based on its physical mechanism, inherent trade-offs remain in terms of material properties and structural design. Piezoresistive sensors offer sensitivity (e.g., MXene aerogel) and wide range (e.g., ILs-MWCNTs-PUs), but are limited by thermal and external interferences. Capacitive sensors provide fast response time (e.g., AgNWs/PVDF-HFP <58 ms), yet suffer from environmental influences and crosstalk. Piezoelectric/triboelectric devices demonstrate ultrafast response (e.g., β-glycine-gelatine, 1 ms) and self-powered capability for dynamic sensing, but face interfacial and internal degradation, affecting long-term durability. Triboelectric sensors are also highly sensitive to humidity. These limitations highlight the urgent need for cross-mechanism collaborative design and novel material strategies.

## 3. Multifunctional Flexible Tactile Sensors

With the rising demand for smart wearable devices and human–machine interaction, single-function tactile sensors are insufficient for complex scenarios. Thus, multifunctional flexible tactile sensors have become a research focus. These sensors can simultaneously detect various stimuli, including pressure from different directions, sliding touch, temperature, and humidity, significantly expanding their application range. This section summarizes recent advancements in multifunctional flexible tactile sensors concerning structural design and material selection.

### 3.1. Structure Design

**Figure 8:**
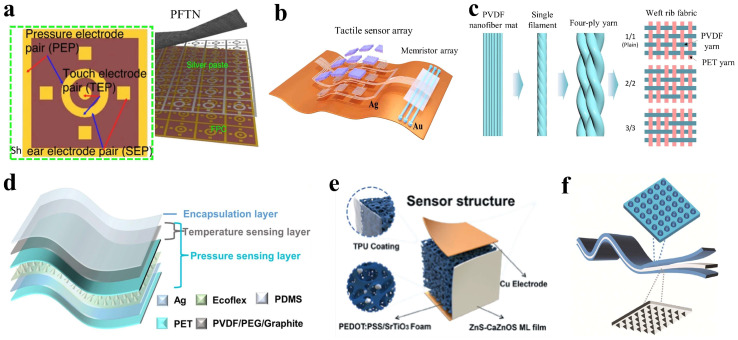
Structural design of multifunctional tactile sensors. (**a**) Schematic structure of the E-skin comprising a sensing array. Reprinted with permission from [91]. Copyright 2024 Springer Nature. (**b**) Schematic diagram of a sensing system with in-plane sensors and memristors. Reprinted with permission from [92]. Copyright 2024 Elsevier. (**c**) Schematic diagram of the woven structures for textile electronics. Reprinted with permission from [93]. Copyright 2022 Springer Nature. (**d**) Structural diagram of the temperature–pressure-integrated sensor. Reprinted with permission from [94]. Copyright 2024 Elsevier. (**e**) Schematic diagram of the optical–thermal-integrated flexible tactile sensor. Reprinted with permission from [95]. Copyright 2024 John Wiley and Sons. (**f**) Schematic diagram of a layered tactile sensor. Reprinted with permission from [96]. Copyright 2024 Royal Society of Chemistry.

The structural design for multifunctionality focuses on integrating individual sensors and various materials to capture multiple types of information. Zhang et al. developed an E-skin made up of multiple sensing units with a flexible printed circuit (FPC) [91]. The specific pattern included one common ring electrode, a circular touch detection electrode, four square traction sensing electrodes, and one quadrupole pressure sensor, as shown in Figure 8a. This configuration allows for the detection of diverse external stimuli, enabling complex haptic interactions, such as pinching, spreading, adjusting, and twisting. In addition to specialized patterns, sensor arrays are also employed in structural designs. Huang et al. fabricated a smart system that uses a broad-range homomorphic sensor array and memristor array, as shown in Figure 8b [92]. This system provides high linear sensitivity and resolution for capturing signals from external pressure stimuli, enabling effective data acquisition and precise classification of human respiratory states. Kim et al. fabricated a woven device using PVDF weft yarns and polyethylene terephthalate (PET) warp yarns in various fabric configurations: 1/1 (plain), 2/2, and 3/3 ribbed styles, as shown in Figure 8c [93]. They utilized the 2/2 ribbed fabrics in textile electronics to effectively monitor diverse physiological motions, such as bending, twisting, crumpling, walking, and running through distinguishable electrical signals.

On the other hand, incorporating insensitive sensing materials and microstructures in a three-dimensional layered method is predominant for multifunctional sensing. Ji et al. designed a temperature–pressure-integrated tactile sensor that combines thermo-resistive and contact-electrical effects to detect body temperature and physiological movements simultaneously, as shown in Figure 8d [94]. In the sensor, different functional layers are stacked to convert temperature and pressure stimuli into two independent output signals, achieving simultaneous detection without signal crosstalk. Xu et al. developed an opto-thermal flexible tactile sensor for liquid recognition [95]. The sensor used PEDOT:PSS/SrTiO_3_ thermoelectric foam and ZnS-CaZnOS mechanoluminescent films, as illustrated in Figure 8e. This design enables the simultaneous detection of thermal and optical changes with minimal interference. Yin et al. [96] achieved high performance in detecting thermal and optical variations with low crosstalk by combining coupled pyramid and dome microstructures, as shown in Figure 8f, enabling multidirectional force detection for sensitive monitoring of human motion.

### 3.2. Material Selection

**Figure 9:**
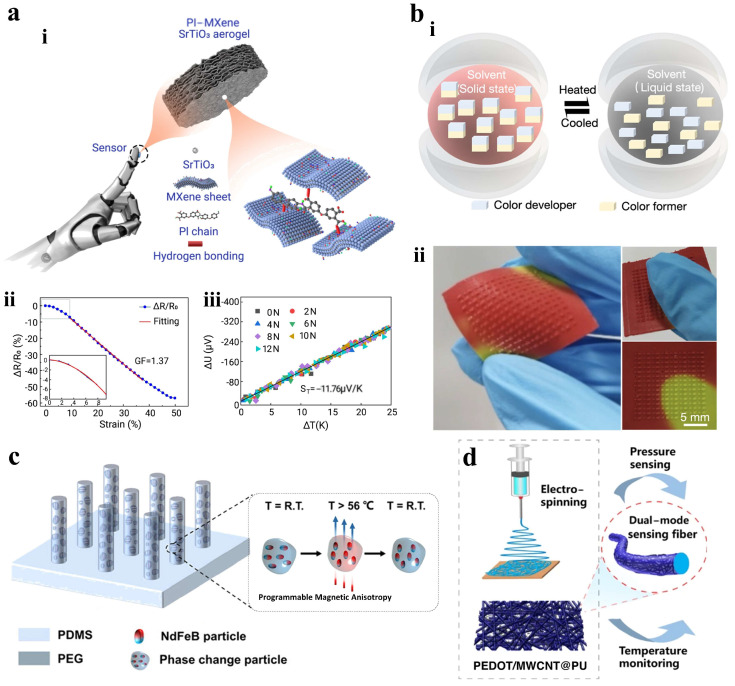
Material selection for multifunctional tactile sensors. (**a**) (**i**) Schematic diagram of a flexible tactile sensor based on PI-MXene/SrTiO_3_ aerogel. (**ii**) Resistive changes to compressive strain. (**iii**) Thermoelectric signals to temperature. Reprinted with permission from [97]. Copyright 2024 Elsevier. (**b**) (**i**) Schematic diagram of the thermochromic temperature sensing. (**ii**) Photos of the visual temperature sensor. Reprinted with permission from [98]. Copyright 2023 American Chemical Society. (**c**) Schematic diagram of the magnetic micropillar arrays and its working principle. Reprinted with permission from [99]. Copyright 2024 American Chemical Society. (**d**) Schematic diagram of the PEDOT/MWCNT@PU ma for pressure and temperature sensing. Reprinted with permission from [100]. Copyright 2023 Springer Nature.

Multifunctional sensing often involves selecting materials with various sensing capabilities. Deng et al. designed a polyimide (PI)-MXene/SrTiO_3_ nano-composite aerogel with a laminated porous structure. The PI substrate provides stability, MXene enables pressure sensing, and the MXene/SrTiO_3_ composite facilitates thermoelectric and infrared responses, as shown in Figure 9a [97]. This sensor can simultaneously detect force and temperature while also sensing infrared radiation, offering precise haptic feedback for robotic manipulators. Additionally, visual sensing can enhance temperature detection. Li et al. developed a tactile sensor using thermochromic microstructures for simultaneous pressure and temperature measurement, as shown in Figure 9b [98]. This sensor reduced interference and simplified design to promote cost-effective multifunctional sensor fabrication.

Magnetic soft materials provide an innovative method for multifunctional sensing. Bao et al. fabricated a self-powered human–machine interaction system using magnetic micropillar arrays with anisotropic magnetization directions [99]. This setup comprises programmed magnetic micropillar arrays and conductive coils, as shown in Figure 9c. The sensor induced electric potentials in response to external magnetic field changes, effectively enabling the detection of sliding touch direction in flexible electronics. Additionally, composite fiber materials are durable and suitable for continuous signal monitoring. Luo et al. employed electrospinning PU nanofibers with MWCNTs to establish conductive paths, as shown in Figure 9d [100]. A layer of PEDOT was then applied to the PU nanofibers to create a composite sensor. The PEDOT/MWCNT@PU mat-based sensor continuously monitored physiological signals related to respiration and pulse activity from the carotid and radial arteries while exhibiting thermoelectric properties for temperature sensing, which has potential applications in wearable monitoring and E-skins.

By integrating soft computing algorithms like neural networks and fuzzy logic with eddy current detection, informative features can be extracted for near real-time defect classification and evaluation. This approach is particularly effective in detecting early-stage delaminations in conductive composites (e.g., CFRP), improving both intelligence and accuracy [101,102]. Moreover, this combination enhances the performance of non-destructive testing technologies and provides an efficient solution for defect detection in composite materials, making it suitable for structural health monitoring of aerospace, automotive, and wind-energy components. Additionally, finite element models of eddy current fields enable prediction of probe impedance amplitude/phase changes caused by cracks, delaminations, and lift-off/geometry variations, thereby improving detection accuracy. This framework not only advances ECT techniques but also supports flexible/conformable eddy current probe arrays for long-term stability monitoring on complex geometries, improving sensor reliability in harsh environments.

## 4. Tactile Sensor Applications

Flexible tactile sensors are essential for intelligent robots, offering high sensitivity, bendability, and adaptability to various surfaces. Recent advancements in materials science and microelectronics have enhanced their applications and improved the flexibility and reliability to current challenges. In health monitoring, flexible tactile sensors enable personalized medicine and rehabilitation by tracking physiological parameters and motion data. In human–machine interaction, they enhance augmented reality (AR) and virtual reality (VR) systems with tactile feedback capabilities.

### 4.1. Health Monitoring

With the rise of chronic diseases, such as hypertension and diabetes, there is growing emphasis on medical care and long-term patient monitoring. Flexible tactile sensors can be embedded in the wrist and chest to monitor blood pressure, pulse, and respiratory rate. These sensors are categorized into two detection modes: in vivo sensing and ex vivo sensing.

**Figure 10:**
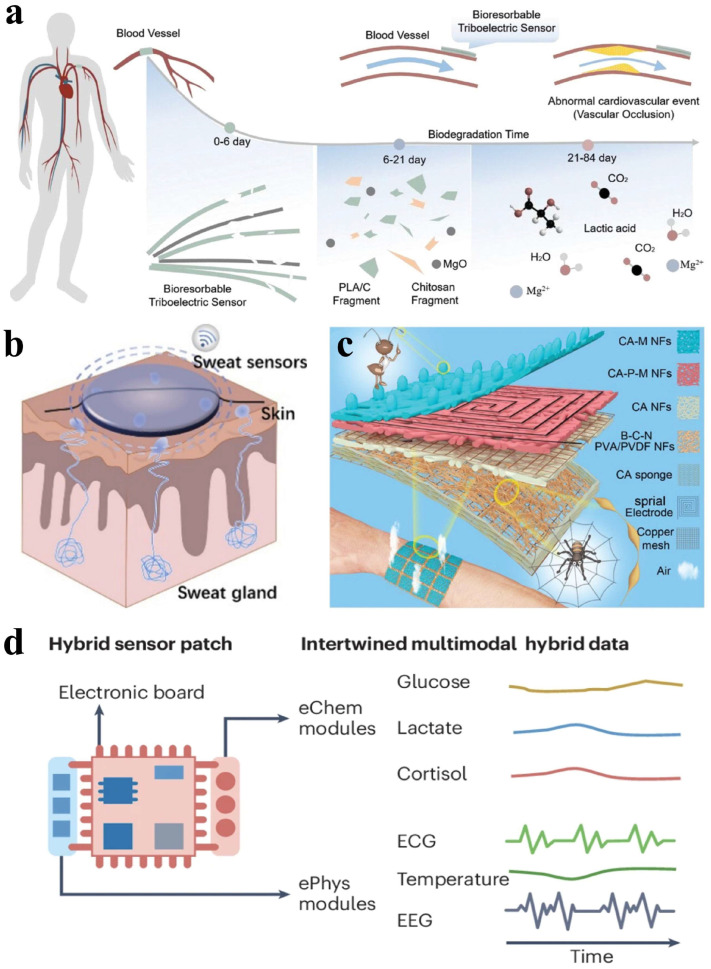
Health monitoring. (**a**) Schematic illustration of the biodegradable and self-powered pressure sensors. Reprinted with permission from [104]. Copyright 2021 John Wiley and Sons. (**b**) The hydrogel sweat sensor on skin. Reprinted with permission from [109]. Copyright 2024 American Chemical Society. (**c**) Nanofiber-based multifunctional sensors. Reprinted with permission from [113]. Copyright 2021 John Wiley and Sons. (**d**) Schematic illustration of a hybrid multifunctional sensor patch. Reprinted with permission from [114]. Copyright 2024 Springer Nature.

In vivo sensing is often used in implantable biomedical devices to monitor biological metabolites, stimulate nerves, detect signals, restore body functions, and deliver drugs [103]. Wang et al. reported a biodegradable self-powered pressure sensor that converts environmental pressure into electrical signals for postoperative cardiovascular care, as shown in Figure 10a [104]. This sensor has two triboelectric layers that generate voltage output upon contact under external force. The sensor captures kinetic energy from the triboelectric effect of degradable materials while demonstrating excellent sensitivity and durability. It features antibacterial performance with a lifespan of five days and a degradation time of 84 days, effectively preventing wound infections. Rogers et al. developed a wireless microfluidic sensing system that operates reliably with mobile terminal readings [105]. This sensor measures blood flow using a heater and thermistor, connecting to the skin via a compact Bluetooth module for data reading and display on mobile devices. Previous studies have shown the effectiveness of electrical impedance tomography (EIT) in tumor detection, as tumor tissue exhibits distinct electrical conductivity and impedance compared to normal tissue [106]. By utilizing highly sensitive flexible tactile sensors, these impedance changes can be accurately measured for early lesion detection [107]. This technology offers real-time monitoring capability and good wearability, highlighting its potential for health monitoring and remote diagnosis.

Ex vivo sensing primarily detects physiological information from the body. Inspired by bionics, integrating sensor devices with flexible substrates has garnered attention for detecting various physiological signals such as muscle and joint movements, temperature changes, and sweating. The weak nature of such human signals necessitates highly sensitive sensors, demanding excellent robustness [108]. Wang et al. presented a wearable strain sensor for real-time sweat monitoring, as shown in Figure 10b [109]. The hydrogel-based sensor absorbs sweat, with its swelling being recorded and analyzed to track sweat volume in real time. Deng et al. introduced a highly sensitive patch-type piezoresistive sensor for monitoring physiological signals [110]. Inspired by rose petal microstructures, this sensor has a layered design of nanofiber films and dome-shaped interlocking structure. The patch sensor had exceptional mechanical flexibility and electrical performance. Moreover, the potential biophysical effects of flexible sensors in direct contact with human tissue have been evaluated, including their electromagnetic interactions and thermal responses. Recent research provided data on electromagnetic behavior and temperature response of flexible sensors in human contact areas to address issues like electromagnetic radiation and temperature changes [111]. It has been shown that current flexible sensors can effectively manage the specific absorption rate (SAR) through optimized design and material selection, while maintaining safe temperature levels. Emphasis has been placed on the use of biocompatible materials (e.g., MXene, PEDOT) and advanced thermal management strategies to reduce interference, thereby ensuring that such sensors do not pose additional electromagnetic hazards or cause excessive heat under operational conditions.

Wearable bioelectronic sensors are used for health monitoring, not only by replicating common functions like human skin, but also detecting additional physical or chemical parameters, which enables comprehensive health assessments. Human skin lacks humidity receptors, leading to poor humidity perception. A study shows that ant tentacles can sense humidity, odors, sounds, and more [112]. Ouyang et al. designed an antenna on the surface of CA-M NFs (CA and acidified MWNTs are compounded and electro-spun to prepare NFs with ant antenna structure) to replicate ant tentacles as the primary layer for humidity sensing, as shown in Figure 10c [113]. This design increases the contact area between water molecules and NF-based structural layers, enhancing permeation speed with response and recovery times of 16 s and 25 s within a humidity range of 25–85% RH. Additionally, the CA PEDOT MNTs NFs were created from these webs to function as temperature-sensing layers. A hybrid multifunctional sensor can be fabricated to enable simultaneous recording of multiple biophysical and biochemical signals, as shown in Figure 10d. The choice of specific biomarkers and physical parameters depends on the targeted pathophysiological conditions [114]. In addition, the sensor kit can be extended to ultra-sensitive optoelectronic devices to significantly increase the accuracy in detecting weak optical signals and biological data [115]. For example, flexible tactile sensors in combination with optoelectronic devices allow for non-invasive blood oxygen and glucose tracking, a widely studied area in biosensing.

### 4.2. Human Machine Interaction

**Figure 11:**
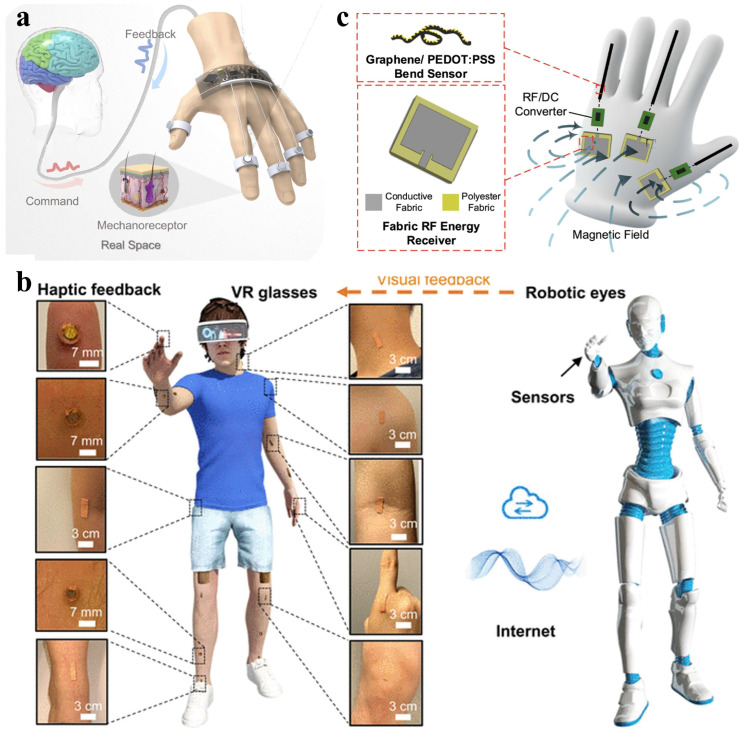
HMI interface-enabled VR/AR and tactile feedback. (**a**) Biological neural network of human finger sensations feeds back into the ATH ring. Reprinted with permission from [116]. Copyright 2022 Springer Nature. (**b**) Closed loop HMI for robot VR applications. Reprinted with permission from [117]. Copyright 2022 Elsevier. (**c**) The conceptual schematic of a glove-based human–machine interface (HMI) system comprising fabric RF energy receivers (antennas). Reprinted with permission from [118]. Copyright 2024 American Chemical Society.

HMI improves the usability and overall experience of systems, software, and digital interfaces by designing user-friendly technologies that cater to cognitive, physical, and emotional needs. Among them, virtual reality (VR) and augmented reality (AR) create immersive experiences by replicating visual and auditory stimuli from the physical world. For effective VR/AR operations and advanced robotics, it is vital to have multi-dimensional sensors along with well-designed tactile feedback loops. Sun et al. proposed an enhanced tactile system for VR applications via incorporating TENG tactile sensors for continuous bending detection, flexible pyroelectric sensors for temperature sensing, eccentric rotating mass vibrators for vibration feedback, and nickel–chromium alloy wires for thermal feedback [116]. All components are integrated into a cohesive tactile loop connected to a wireless IoT module, as shown in Figure 11a. Xu et al. developed a closed-loop HMI for wireless motion capture and tactile feedback, as shown in Figure 11b [117]. This system integrates visual and tactile VR, allowing for remote control of robots through skin-based platforms. Figure 11c shows a glove-based HMI system with three RF energy receivers on the back, each connected to graphene/PEDOT:PSS-coated yarn along the thumb, middle, and little fingers via RF-to-DC rectifiers. This wireless design enables energy-efficient and flexible control for wearable applications [118].

**Figure 12:**
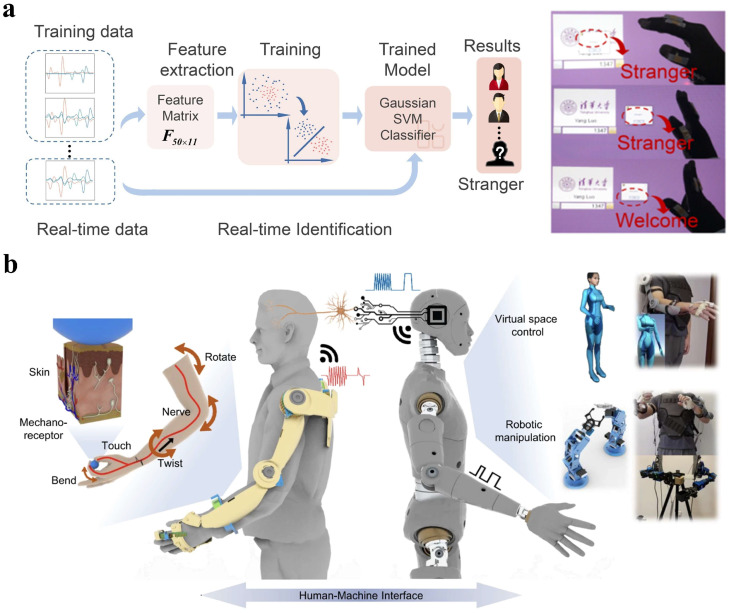
Machine learning-enhanced HMIs. (**a**) Finger sensing of a raw a glove-based multidimensional HMI with user recognition via SVM algorithm. Reprinted with permission from [122]. Copyright 2021 Elsevier. (**b**) Schematic of an exoskeleton sensing system used to implement virtual space and robotic manipulation. Reprinted with permission from [126]. Copyright 2021 Springer Nature.

Intelligent HMIs combine electronic sensors and developed software to enhance system performance by analyzing specific signals [119]. Machine learning algorithms are employed for haptic information processing in object recognition, material sensing, touch modality classification, and HMI applications due to their strong pattern recognition abilities, such as convolutional neural networks (CNNs), support vector machines (SVMs), and k-nearest neighbors (KNN) [120]. Specifically, long short-term memory (LSTM) networks and U-Net models have been widely applied in temporal data analysis and image segmentation tasks. Recent studies have demonstrated the use of LSTM models for predicting current absorption (CA) and achieving efficient energy consumption prediction through precise analysis of sensor timing data. Additionally, the U-Net model has been successfully employed for anomaly detection in thermal imaging, enabling real-time identification of potential system issues [121]. Luo et al. fabricated an SVM-based software platform integrated with a glove-based HMI for user identification, as shown in Figure 12a [122]. Gesture recognition is possible using tactile and force sensors on hands and fingers. However, this can hinder users’ hand tasks. An HMI sensing system mounted on the arm can be developed to accurately recognize gestures via muscle movement signals combined with machine learning algorithms [123]. Meanwhile, real-time monitoring of muscle activity is possible through the integration of electromyography (EMG) sensors, which can provide data for motion pattern recognition [124]. Artificial intelligence algorithms, such as CNN and SVM, can efficiently extract features and identify patterns from EMG signals, enabling the recognition of complex gestures [125]. This AI-enhanced approach can facilitate intelligent health monitoring and human–machine interactions, which further facilitates fast feedback in upper-limb exercise training and rehabilitation processes. Chen et al. proposed a cost-effective triboelectric bi-directional sensor for customizable exoskeletons, capable of monitoring all movable joints in the human upper limbs with low power consumption. It detects movements such as two degree-of-freedom shoulder rotations and wrist twisting and bending, enabling real-time control of virtual characters and robotic arms, as shown in Figure 12b [126]. Flexible tactile sensors play a crucial role in wearable therapy and rehabilitation, especially for real-time monitoring of muscles, joints, and skin. Filippo Lagan et al. combined FEM modeling with artificial intelligence-driven analysis to simulate human motion, while using sensor data to classify and evaluate the motion process [127]. This approach accurately models movements and contacts through FEM, providing more accurate predictions of patient recovery progress and improving individual adaptation.

### 4.3. Environmental Monitoring and Sensing

Advances in flexible tactile sensor technology are expanding their applications from health monitoring and human–machine interaction to a variety of natural environments. These conditions challenge the sensitivity, stability, and adaptability of the sensors while fostering innovation in flexible tactile sensors. In agriculture, intelligent sensors are playing an important role in detecting mineral elements, humidity, temperature, and crop health [128].

**Figure 13:**
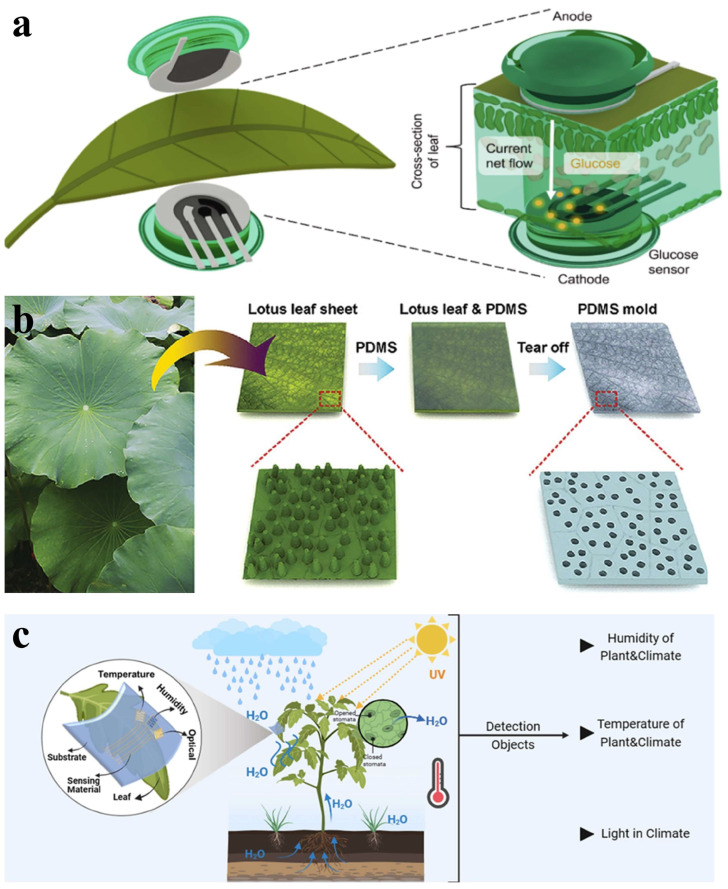
Environmental monitoring and sensing. (**a**) Glucose-selective sensor combined with reverse iontophoresis. Reprinted with permission from [129]. Copyright 2023 Elsevier. (**b**) Schematic of the fabrication process of a PDMS mold with a lotus leaf-like structure. Reprinted with permission from [130]. Copyright 2020 Elsevier. (**c**) Schematic diagram of a plant equipped with a multifunctional wearable sensor for extrinsic microclimate monitoring, where the detection objects included in the plant microclimate include the humidity and microclimate of the plant itself, the temperature of the plant itself, as well as the microclimate and light in the microclimate. Reprinted with permission from [132]. Copyright 2024 John Wiley and Sons.

Perdomo et al. combined a glucose-selective sensor with reverse iontophoresis to enable glucose flow to the measuring electrode (cathode) within 10 min, as shown in Figure 13a [129]. There is an applied current between the anode and cathode of the sensor. The presence of the glucose oxidase-modified electrode facilitates oxygen reaction to yield hydrogen peroxide (H_2_O_2_), allowing quantitative glucose detection through measuring either H_2_O_2_ production or oxygen consumption without harming plant leaves. Li et al. developed a self-powered triboelectric nanogenerator integrated with micro-supercapacitors that captures energy from its environment [130]. It harvests mechanical energy from leaf movements and potential energy from falling raindrops. Notably, this waterproof device uses a lotus leaf bionic structure for self-cleaning capabilities, making it functional in wet outdoor environments or acid rain scenarios, as shown in Figure 13b. The device can work even in wet outdoor environments or acid rain conditions. Tsong et al. employed wearable plant sensors to monitor external microclimates of humidity, temperature, light levels, air movement, and soil moisture, as shown in Figure 13c [131]. This data is crucial for optimizing growing conditions, such as light intensity, temperature control, humidity management, and other vital factors essential for healthy plant development [132].

## 5. Conclusions

### 5.1. Research Gaps and Future Directions

The current deployment of flexible tactile sensors still faces several critical research gaps. While polymer substrates (e.g., PDMS [57,65] and hydrogels [66]) offer flexibility, their vulnerability to environmental factors persists, such as resistance drift in MXene composites under high humidity [58,64]. Microstructural advances have increased sensitivity, but challenges including cycle degradation and structural failure still limit reliability [73]. Multimodal integration remains particularly challenging: current temperature and pressure sensors struggle with interference in health monitoring [94,110], and laboratory metrics are not aligned with real-world industrial or medical needs. Addressing these gaps requires cross-scale approaches, including creating environmentally robust composites, designing fatigue-resistant bio-inspired architectures with self-healing elastomers, leveraging neuromorphic processing frameworks such as memristor-based in-sensor computing [123], and the establishment of standardized protocols for application validation.

### 5.2. Summary and Perspectives

This paper reviews the application of flexible and wearable tactile sensors in intelligent systems, emphasizing their transduction mechanisms and multifunctional sensing technologies. These sensors hold significant potential for health monitoring, human–machine interaction, and environmental surveillance. The discussion encompasses various mechanisms, including piezoresistive, capacitive, piezoelectric, and triboelectric sensors, underscoring how multifunctional sensing capabilities enhance performance.

Despite the promise offered by flexible wearable tactile sensors, several challenges remain unaddressed. Issues such as material durability, systematic integration, reliable data processing frameworks, and varying condition adaptability continue to desire progress. Advancements in developing sensitive and durable materials could significantly improve electrical conductivity, mechanical strength, and flexibility. Moreover, integrating deep learning modules can promote both efficient processing and accurate identification of tactile results. Implementing adaptive learning algorithms may further enable these sensors to dynamically adjust their data processing methods in specific scenarios. Such advancements facilitate smart systems that can more effectively detect diverse stimuli in complex environments, thereby expanding their range of applications.

Future designs will likely focus on creating more miniaturized solutions that promote natural interactions between users and technology. The scope of application for flexible wearable tactile sensors is anticipated to broaden across sectors such as education, entertainment, and industry. This evolution will promote social robots to better comprehend human needs while enhancing comfort. Subsequently, future research should prioritize interdisciplinary collaboration as a catalyst for ongoing innovation within this field.

## Figures and Tables

**Table 1:** Performance comparison of four types of flexible tactile sensors.

Sensor Types	Materials	Sensitivity	Sensing Range	Response Time	Durability (Cycles)	Ref.
Piezoresistive tactile sensor	NC-GF/PDMS	4.2 kPa^−1^ (0.5–25 kPa)	>0.5 Pa	150 ms	10,000	[57]
	MXene aerogel	1900 kPa^−1^	0.0063~0.02 Pa	<100 ms	10,000	[58]
	ILs-MWCNTs-PUs	7.023 kPa^−1^ (0–0.1 kPa)	0.1~420 kPa	60 ms	80,000	[65]
Capacitive tactile sensors	MXene/MWCNTs	4.2 kPa^−1^ (0–1 kPa)	0~700 kPa	46 ms	4500	[71]
	AgNWs/PVDF-HFP	48.57 kPa^−1^ (0–1 kPa)	>5.5 mg	<58 ms	>3000	[73]
	Polyester fiber/carbon	1.47 kPa^−1^ (0.1–10 kPa)	0.1~100 kPa	~35 ms	4000	[74]
Piezoelectric tactile sensor	P(VDF-TrFE)/ZnO	8.30 mV/kPa	0~30 N	~5 ms	>10,000	[83]
	β-glycine-gelatine	41.3 mV/kPa	2.5~55 kPa	1 ms	/	[90]
Triboelectric tactile sensor	BaTiO_3_/PDMS	3.71 V/kPa (0–15 kPa)	0.1~100 kPa	/	5000	[88]
	Carbon quantum dot-reinforced hydrogel	5.16 V/kPa (0–15 kPa)	1~25 N	210 ms	>7500	[89]

## Data Availability

No new data were created or analyzed in this study. Data sharing is not applicable to this article.
